# Mesenchymal stem cells-derived extracellular vesicles in acute respiratory distress syndrome: a review of current literature and potential future treatment options

**DOI:** 10.1186/s40169-019-0242-9

**Published:** 2019-09-12

**Authors:** Trushil G. Shah, Dan Predescu, Sanda Predescu

**Affiliations:** 1 Department of Internal Medicine, Pulmonary, Critical Care and Sleep Medicine, Rush University Medical Center, 1750 W Harrison St. 1535 JS, Chicago, IL 60612 USA; 2 Division of Pulmonary and Critical Care Medicine, University of Texas Southwestern, Dallas, TX USA

**Keywords:** Mesenchymal stem cells, Extracellular vesicles, Acute respiratory distress syndrome, Acute lung injury

## Abstract

Acute respiratory distress syndrome (ARDS) is a life-threatening inflammatory lung condition associated with significant morbidity and mortality. Unfortunately, the current treatment for this disease is mainly supportive. Mesenchymal stem cells (MSCs) due to their immunomodulatory properties are increasingly being studied for the treatment of ARDS and have shown promise in multiple animal studies. The therapeutic effects of MSCs are exerted in part in a paracrine manner by releasing extracellular vesicles (EVs), rather than local engraftment. MSC-derived EVs are emerging as potential alternatives to MSC therapy in ARDS. In this review, we will introduce EVs and briefly discuss current data on EVs and MSCs in ARDS. We will discuss current literature on the role of MSC-derived EVs in pathogenesis and treatment of ARDS and their potential as a treatment strategy in the future.

## Acute respiratory distress syndrome (ARDS)

ARDS is a life-threatening inflammatory lung condition characterized by recognizable diffuse alveolar damage and lung injury [1, 2]. ARDS affects 200,000 Americans annually and carries a high mortality rate of 30–50% [3]. Out of patients who survive, significant morbidity occurs from neuromuscular weakness, neuropathy, myopathy, residual lung fibrosis and cognitive issues [4]. Even 5 years after ARDS, some of these morbidities persist leading to increased healthcare utilization and cost [4]. ARDS is classified as mild, moderate and severe, based on the degree of hypoxemia as gauged by PaO_2_/FiO_2_ ratio, where mild includes what was previously referred to as acute lung injury (ALI) [3]. Even to date, many studies use ALI interchangeably for ARDS. The current definition of ARDS is clinical, based on chest X-ray finding of bilateral infiltrates, the timing of initial injury and measure of hypoxemia (PaO_2_/FiO_2_), which applies to heterogeneous, complex conditions with potentially different mechanisms [3]. Underlying causes of ARDS vary, but regardless of underlying causes (i.e., pneumonia, sepsis, pancreatitis, trauma), clinically, patients seem to follow a similar pattern of lung injury [3]. The accompanying lung injury is caused by activation of intense pulmonary and systemic inflammation with release of pro-inflammatory cytokines leading to endothelial and epithelial damage and accumulation of proteinaceous exudate in the alveoli [5].

Interestingly, clinical outcomes of ARDS are variable in patients, where some recover completely, some survive with a permanent decrease in lung function, while others die [4, 6]. Currently, our efforts for management and treatment of this deadly disease remain supportive with efforts to reduce ventilator-induced lung injury and allow endogenous repair process to help with recovery from lung injury. While pathologically, we often encounter different stages of ARDS from exudative to fibroproliferative stages, clinically these are not as relevant, as often the lung injury (exudative phase) and repair coexist [5]. Also, the repair process is not uniform across all patients. While some patients recover lung function completely, others have decreased lung function where part of the lung is replaced by fibrotic tissue [7]. Understanding the differences in the recovery process and why some patients have more fibrosis than others will help identify new frontiers for treatment strategies. Currently, we do not have any effective curative treatment strategy to reverse lung inflammation in ARDS [8]. Extracellular vesicles (EVs) are increasingly recognized to play an important role in the pathogenesis of ARDS. Based on upcoming literature, EVs have the potential to work as biomarkers as well as therapy for ARDS. Mesenchymal stem cells (MSCs) due to their immunomodulatory, antimicrobial and anti-inflammatory functions are being actively studied with multiple preclinical studies supporting their use [9]. Currently, MSCs are being investigated as a therapy for ARDS in phase 1 and phase 2 clinical trials. In this review, we will introduce EVs, discuss the unique properties of MSCs and their potential for ARDS treatment and then discuss the role of MSCs-derived EVs in the recovery and treatment of ARDS. Finally, we will discuss why MSCs-derived EVs may be preferable to MSCs for the treatment of ARDS and future possibilities in this area.

## Extracellular vesicles (EVs)

EVs, once considered cellular debris, are increasingly recognized as mediators of intercellular communication in health and disease. EVs comprise exosomes, microvesicles (MVs) and apoptotic bodies [9]. Exosomes are small vesicles of 20–100 nm size with endosomal origin. The exosomes exist intracellularly within multivesicular bodies which fuse with the cell membrane and release the exosomes into extracellular space [9]. Exosomes are rich in proteins like heat shock proteins, annexins, cytoskeletal proteins, signal transduction proteins and multivesicular body synthesis proteins. MVs, in contrast to exosomes, form directly by budding from the cell membrane and are 100 nm to 1000 nm in size [9]. They are abundant in selectins, integrins, CD-40, phosphatidylserine and metalloproteinases. Apoptotic bodies are fragments of dying cells which form and are released in the extracellular space by budding of the plasma membrane during the apoptotic process. They are irregular in shape and are between 50 and 4000 nm in size. [9] They are rich in DNA and histones.

EVs can originate from different cell types in the human body and mainly function as a messenger. EVs carry membrane proteins, cytosolic proteins, transcription factors, mRNA, rRNA, miRNA and various signal transduction molecules. EVs, secreted or shed allow intercellular information exchange via different mechanisms such as internalization, ligand-receptor interaction, secreted factors and fusion-mediated transfer of surface receptors, to name a few [10]. Thus, the presence of EVs by themselves in health and disease may be detrimental or beneficial depending on the cell of origin, the cargo they carry and the information they relay.

## EVs in ARDS

**Fig. 1:**
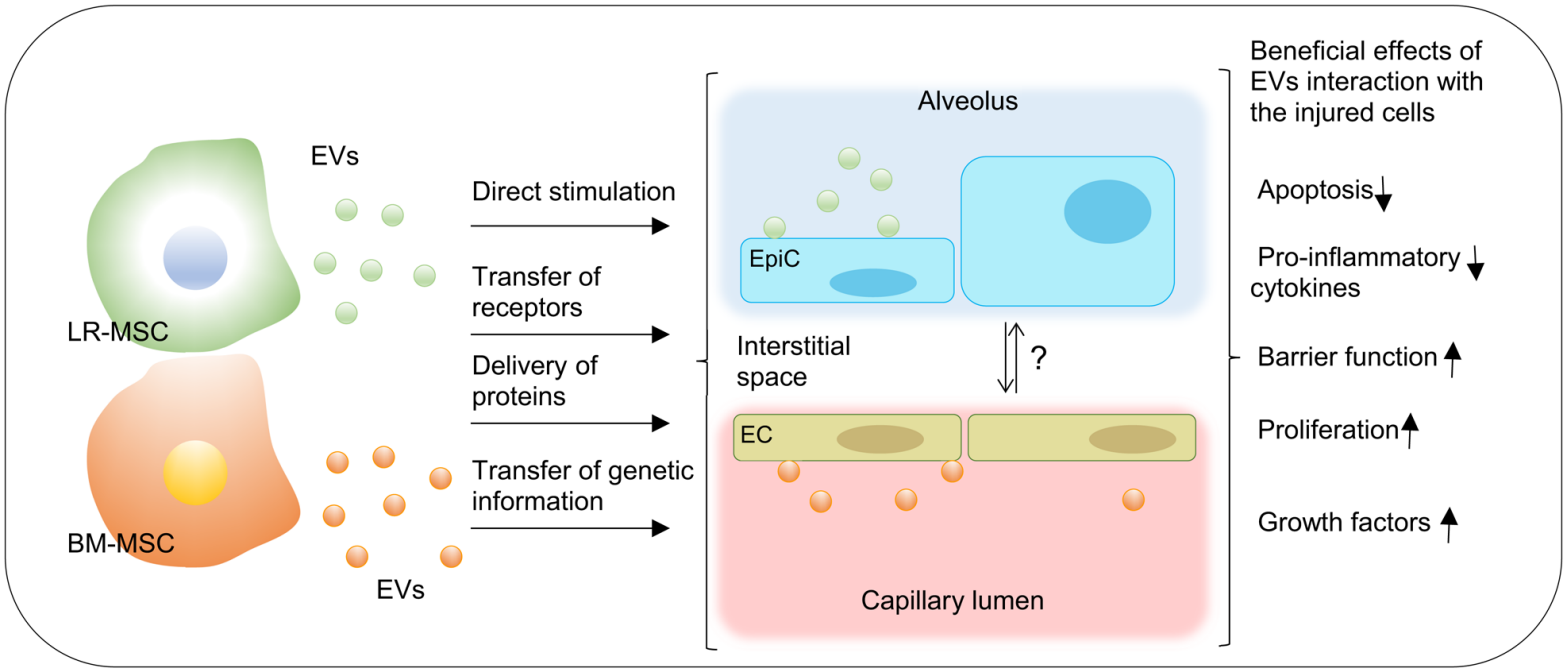
Effects of MSC-derived EVs in ARDS. After an injury, MSCs are mobilized from their specific niches [bone marrow (BM), adipose tissue, etc.], and transit via the blood to the site of injury. They participate in the tissue repair process, either directly or in a paracrine manner by releasing EVs. Also, under disease conditions, LR-MSCs migrate into the alveolar space and function hand-in-hand with the type II epithelial cells in a paracrine manner by releasing EVs to achieve epithelial repair following injury. The EVs interact/are internalized into the target cells such as endothelial cells, monocytes, macrophages, and neutrophils. The net effects are decreased apoptosis, reduced levels of pro-inflammatory cytokines, improved barrier function and increased proliferation. The cellular crosstalk between the airway epithelium and vascular endothelium and how the behavior of endothelial and epithelial cells is affected by the EVs interaction are not understood. (LR-MSC: lung resident MSC; BM-MSC: bone marrow-derived MSC; EpiC: epithelial cells; EC: endothelial cells)

In the last decade, interest in exploring the role of EVs in pathogenesis and treatment of ARDS has significantly increased leading to a better understanding of their role in this inflammatory lung disease. Multiple studies have shown that EVs derived from the endothelium, epithelium, neutrophils, platelets, mononuclear cells and macrophages play a significant role in the development of lung injury [11–15]. Both alveolar and intravascular EVs of different cellular origins are implicated in the pathogenesis of ARDS (Fig. 1). Indeed, intravenous and intratracheal injection of blood EVs from lipopolysaccharide (LPS)-treated rats to naïve rats induces ARDS [16] LPS-treatment causes a significant increase in myeloperoxidase, tumor necrosis factor alpha (TNF-α), interleukin-1β, in both bronchoalveolar lavage (BAL) fluid as well as plasma of naïve rats [16]. Also, EVs derived from platelets and red blood cells are present in stored blood and induce lung injury, supporting their role in the development of transfusion- associated ALI [17, 18]. Various other approaches have been explored to ameliorate this EV-mediated lung damage including the use of simvastatin, magnolol, and removal of endothelial-derived EVs by continuous venovenous hemofiltration [19–21]. Newer studies show that EVs derived from endothelial progenitor cells and MSCs can help ameliorate ALI [22]. We will focus on favorable properties of MSCs and their derived EVs for treatment in ARDS and future directions.

## MSCs in ARDS

MSCs are multipotent progenitor cells with immunomodulatory properties which exist in nearly all forms of post-natal organs and tissues [9]. Their anti-inflammatory and immunosuppressive properties play an important role in pathogenesis and repair in multiple inflammatory diseases. Beneficial effects of MSCs are due to immune modulation, enhanced bacterial clearance, resolution of inflammation and restoration of capillary barrier function [23]. Moreover, growing evidence suggests that the MSCs interact with the lung microenvironment and their effects are mediated primarily in a paracrine manner by releasing MPs rather than local engraftment [24–27]. Previous studies have shown that MSCs expedite and promote recovery in models of lung injury like *Escherichia coli* endotoxin-induced lung injury, hypoxia-induced lung injury, systemic sepsis, LPS-induced lung injury and ventilator-induced ALI [28–32]. In these models, the mechanism of the repair process was attributed to inhibiting TNF-α release, enhancing IL-10 secretion, decreasing IL-6 levels, increasing keratinocyte growth factor (KGF), overexpression of angiopoietin-1, reprogramming of macrophage function and secretion of antimicrobial peptides [28, 32, 33]. Based on these immunomodulatory properties and beneficial effects of MSCs in preclinical studies, MSCs are now in early-phase clinical testing in patients with ARDS. To date, two phase 1 clinical trials of bone marrow and adipose tissue-derived MSCs have shown no adverse effects in human patients [34, 35].

These therapeutic effects of MSCs are exerted at least in part by their secretome which includes EVs and soluble paracrine factors [36]. MSC-derived EVs are increasingly studied for their potential for the treatment of several diseases.

## MSCs-derived EVs

Multiple groups have reported the therapeutic potential of MSC-derived EVs. These EVs can be derived from endogenous MSCs or can be administered exogenously after being released in the conditioned growth medium by cultured human MSCs. They have the potential to serve as biomarkers as well as treatment options for ARDS in the future. Even with normal recovery from ARDS, MSC-derived EVs seem to play a critical role in the recovery process [37]. In previous work from our lab, we identified a subset of EVs from endogenous bone marrow-derived MSCs in the blood of long-term ARDS survivors which confer a survival advantage to these patients [37]. These EVs express Runx-1 transcription factor and TGFβ receptor-1 which help to facilitate the endothelial cell proliferation and recovery. We have found that the protein expression pattern of two Runx1 isoforms (p52 and p66) is critical for the ARDS outcome: p52 expression is continuous, while the p66 is short-lived. A high ratio of Runx1p66/Runx1p52 is strongly associated with long-term survival in ARDS and serves as a biomarker to distinguish survivors from non-survivors, regardless of disease severity or/and cause (direct or indirect injury) of ARDS [37].

Multiple groups have studied and found a therapeutic advantage of administration of MSC-derived MVs by inhalational or intravascular routes through different mechanisms. Zhu et al. [38] showed that intratracheal instillation of MSC-derived MVs in *Escherichia coli* endotoxin-induced lung injury reduced extracellular lung water, decreased pulmonary edema and lowered lung protein permeability. These MVs also decreased the influx of neutrophils and reduced macrophage inflammatory protein-2 levels in BAL fluid [38]. Interestingly, the same group has demonstrated that one of the therapeutic mechanism of MSCs in ALI is mediated through increased production of KGF [39]. Authors have demonstrated that pretreatment of MSCs with KGF siRNA partially eliminated the therapeutic effect of MSC-derived MVs. They concluded that this therapeutic effect is mediated by the transfer of KGF mRNA from MVs to the alveolar epithelium [38]. MVs derived from human MSCs administered to mice injured with bacterial pneumonia decreased the influx of inflammatory cells, cytokines, protein and bacteria [40]. CD44 receptors mediated the uptake of MVs and they were essential for the therapeutic effects of MVs [40]. In LPS-induced ALI mouse model, Islam et al. [41] showed that bone marrow-derived stem cells release mitochondria containing MVs which are then engulfed by injured epithelium improving bioenergetics in the epithelium. The engulfed mitochondrial MVs increased the survival of mice in LPS-induced ALI, and this survival was lost if MSCs contained dysfunctional mitochondria or were depleted of connexin-43 [41]. Morrison et al. [42] showed that MSCs modulate macrophages in ALI by EV-mediated mitochondrial transfer. Tang et al. [43] showed that MSC-derived MVs contain a substantial quantity of angiopoietin-1 and that the immunomodulatory properties of MSCs on macrophages are partly mediated by transferring angiopoietin-1 mRNA to macrophages. In a cecal ligation model of mouse sepsis, Chang et al. [44] showed that intravenous administration of MSC-derived exosomes significantly decreased albumin level in BAL fluid. They also decreased the levels of TNF-α, nuclear factor-kappa B (NF-kappa B), matrix metallopeptidase 9 (MMP-9) and IL-1β in the lung parenchyma. In the same study, apoptotic MSC-derived MVs were less effective in the mentioned beneficial effects.

Few studies have shown beneficial effects of MSC-derived MVs on human microvascular endothelial cells in culture. Wang et al. [45] showed that MSC-derived exosomes improve LPS-induced permeability in part via hepatocyte growth factor, by decreasing endothelial cell apoptosis, increasing IL-10 production and decreasing IL-6 production. Hu et al. [46] showed that similar to mouse models, MSCs-derived MVs restored tight junctional integrity and endothelial permeability in IL-1β, TNF-α and IFN-γ pretreated human lung microvascular endothelial cells. Anti-CD44 and angiopoietin-1 siRNA pretreatment eliminated the therapeutic effects of the MVs suggesting that CD-44 is essential for uptake and angiopoietin-1 mRNA transfer as a mechanism of repair.

Importantly, MSCs can be pretreated to enrich the expression of a subset of MVs which can increase their therapeutic potential. In a study by Monsel et al. [40] pretreatment of MSCs with a toll-like receptor 3 agonist enhanced the therapeutic effect of MSC-derived MVs, whereas, Park et al. [47] observed increased antimicrobial activity in ex vivo perfused human lung injured with severe *Escherichia coli* pneumonia when MSCs were pretreated with the toll-like receptor 3 agonist. Similarly, Song et al. [48] showed that pretreatment with IL-1β enhanced immunomodulatory effects of MSCs partially through an exosome-mediated transfer of miR-146a.

While the bone marrow is the best-studied stem cell niche, there is also a specialized lung resident stem cell niche (LR-MSCs) comprising cells with self-renewing, clonogenic and multipotent in vitro properties, possibly committed to endogenous lung tissue repair and regeneration [49, 50]. Evidence indicates that in disease, the LR-MSCs migrate from their in-tissue niche into the alveolar space and are abundant and recoverable from the BAL fluid [50, 51]. The LR-MSCs function hand-in-hand with the type II pneumocytes to achieve epithelial repair following injury; similar to bone marrow-derived MSCs, the effects of the LR-MSCs are mediated mainly via paracrine signaling [i.e., release of soluble signaling and immuno-modulatory molecules as well as EVs], rather than local engraftment, Fig. 1 [24, 52, 53]. While this crosstalk can be achieved through the release of small molecules (cytokines, chemokines, growth factors, etc.) leading to changes in endothelial cells behavior/activation [54], whether the BAL-EVs interaction with the alveolar epithelial cells affects endothelial cells behavior is not known.

## Potential benefits of MSC-derived EVs over MSCs

Currently, few phase 1 and phase 2 clinical trials are ongoing to evaluate the utility of administration of MSCs in ARDS [55]. While MSCs have shown promise in the treatment of multiple diseases due to their therapeutic potential exerted by their immunomodulatory properties, concern for potential tumor formation remains [36]. A meta-analysis of clinical trials has shown that administration of MSCs is safe but long-term data are lacking [56]. Compared to MSCs, MSC-derived EVs would be inherently safer for intravenous administration to patients and the risk of tumor formation would be much lower. Also, generally speaking, as the MSC-derived EVs do not carry MHC 1 and 2 class antigens they would be less immunogenic. Modification and enrichment of a particular subset of EVs by pretreatment of MSCs could also be used to increase their potency. The phenotype of MSCs and their derived EVs may be different depending on their growth stages [37]. Thus, their growth stage and phenotype at the time of isolation need to be considered when the therapeutic efficiency of the MSCs is investigated.

## Future research

Genetic modification of MSC-derived EVs with differently targeted cargos like mRNA, cytokines, receptors, ligands, and proteins could help further engineer more potent subsets of EVs with different therapeutic effects [57]. Modified MSC-derived EVs with the potential of providing effective and clinically safe therapeutic approaches may translate into novel strategies to effectively treat ARDS, and thus, it could be the treatment choice of the future. Studies to identify sub-phenotype(s) of EVs with a disease-specific cargo, possibly associated with survival and long-term clinical trials are needed to explore the beneficial effects of EVs in humans further.

## Conclusion

MSC-derived EVs are increasingly recognized for their role in mitigation and repair of lung injury in ARDS. Further human studies are needed in this regard. Studies of EVs, their poorly understood biochemical cargo, functions and mechanisms with which they interact with the damaged lung cells are necessary. Investigations into these facets of the biology of EVs will advance the field and could lead to the clinical translation of EVs-based therapeutics, a more suitable alternative to any stem cell therapy and will even aid in the engineering of synthetic EVs.

## Data Availability

Not applicable.

## Abbreviations

ALI: acute lung injury
ARDS: acute respiratory distress syndrome
BAL: bronchoalveolar lavage
EVs: extracellular vesicles
KGF: keratinocyte growth factor
LR-MSCs: lung resident MSCs
LPS: lipopolysaccharide
MSCs: mesenchymal stem cells
MPs: microparticles
